# Supplementary material for: Understanding Failure and Improving Treatment Using HDAC Inhibitors for Prostate Cancer
Source: Biomedicines. 2020 Jan 30;8(2):22. doi: 10.3390/biomedicines8020022 (PMC7168248; doi:10.3390/biomedicines8020022)
Supplement: Supplementary file 1 [file biomedicines-08-00022-s001.pdf]

Supplementary Table S1. Comparison of microarray (Oncomine database) gene expression levels of HDAC1 (a), HDAC2 (b), HDAC3 (c), HDAC4 (d), HDAC6(e), HDAC9 (f & g) and HDAC10 (h) between prostate gland and prostate carcinoma or prostate adenocarcinoma has been depicted (19).

(a)

Study	Log2 median-centered ratio	Prostate gland	Prostate carcinoma	Fold change	P-value	Over-expression Gene Rank
Tomlins et al	Maximum	1.145	3.234	2.209	9.78E-5	476 (in top 5%)
	90th percentile	0.605	2.449			
	75th percentile	0.368	2.049			
	Median	-0.165	0.892			
	25th percentile	-1.004	-0.219			
	10th percentile	-1.357	-0.792			
	Minimum	-1.575	-1.31			
	Number of samples	23	30			
Lapointe et al	Maximum	1.168	1.604	1.379	1.96E-7	411 (in top 5%)
	90th percentile	0.342	0.952			
	75th percentile	0.143	0.803			
	Median	-0.13	0.351			
	25th percentile	-0.344	-0.026			
	10th percentile	-0.527	-0.246			
	Minimum	-0.582	-0.79			
	Number of samples	39	61			
Grasso et al	Maximum	0.234	1.204	1.251	8.54E-8	344 (in top 2%)
	90th percentile	0.187	0.929			
	75th percentile	0.086	0.596			
	Median	0.014	0.188			
	25th percentile	-0.059	0.048			
	10th percentile	-0.231	-0.081			
	Minimum	-0.399	-0.228			

	Number of samples	28	59			
Yu et al	Maximum	3.017	3.808	1.423	5.82E-7	106 (in top 2%)
	90th percentile	2.893	3.403			
	75th percentile	2.852	3.244			
	Median	2.538	2.892			
	25th percentile	2.303	2.778			
	10th percentile	1.84	2.674			
	Minimum	1.739	2.391			
	Number of samples	23	65			
Welsh et al	Maximum	3.416	4.731	1.599	1.84E-7	98 (in top 2%)
	90th percentile	3.416	4.321			
	75th percentile	3.297	4.168			
	Median	3.205	3.79			
	25th percentile	3.135	3.602			
	10th percentile	2.691	3.42			
	Minimum	2.691	3.225			
	Number of samples	9	25			
Magee et al	Maximum	2.622	3.984	1.355	0.025	318 (in top 6%)
	90th percentile	2.622	3.984			
	75th percentile	2.622	3.308			
	Median	2.571	2.888			
	25th percentile	2.522	2.727			
	10th percentile	2.45	2.293			
	Minimum	2.45	2.293			
	Number of samples	4	8			

(b)

Study	Log2 median-centered ratio	Prostate gland	Prostate carcinoma	Fold change	P-value	Over-expression Gene Rank
Tomlins et al	Maximum	1.06	2.754	3.213	1.19E-4	498 (in top 5%)
	90th percentile	1.06	2.493			
	75th percentile	0.036	1.284			
	Median	-1.228	0.823			
	25th percentile	-1.575	0.363			
	10th percentile	-1.813	-0.047			
	Minimum	-2.019	-1.559			
	Number of samples	10	20			
Varambally et al	Maximum	4.084	4.492	1.404	9.86E-4	324 (in top 2%)
	90th percentile	4.084	4.492			
	75th percentile	4.084	4.492			
	Median	3.875	4.313			
	25th percentile	3.831	4.179			
	10th percentile	3.391	4.065			
	Minimum	3.391	4.065			
	Number of samples	6	7			

(c) HDAC3

Study	Log2 median-centered ratio	Prostate gland	Prostate carcinoma	Fold change	P-value	Over-expression Gene Rank
Yu	Maximum	-0.319	-0.171	1.120	7.92E-4	717 (in top 9%)
	90th percentile	-0.572	-0.386			
	75th percentile	-0.681	-0.515			
	Median	-0.818	-0.655			
	25th percentile	-0.96	-0.739			
	10th percentile	-1.057	-0.893			
	Minimum	-1.13	-1.314			
	Number of samples	23	65			

(d) HDAC4

Study	Log2 median-centered ratio	Prostate gland	Prostate carcinoma	Fold change	P-value	Over-expression Gene Rank
Arreduoani Prostate	Maximum	-0.902	-0.034	1.340	5.67E-4	415 (in top 3%)
	90th percentile	-0.902	-0.104			
	75th percentile	-0.902	-0.231			
	Median	-1.507	-0.763			
	25th percentile	-1.117	-0.9			
	10th percentile	-1.38	-0.942			
	Minimum	-1.38	-1.07			
	Number of samples	8	13			

(e) HDAC6

Study	Log2 median-centered ratio	Prostate gland	Prostate carcinoma	Fold change	P-value	Over-expression Gene Rank
Holzbeierlein Prostate	Maximum	-0.212	0.004	1.049	0.025	213 (in top 3%)
	90th percentile	-0.212	-0.085			
	75th percentile	-0.212	-0.136			
	Median	-0.267	-0.177			
	25th percentile	-0.282	-0.247			
	10th percentile	-0.282	-0.281			
	Minimum	-0.282	-0.38			
	Number of samples	3	24			

(f) HDAC9

Study	Log2 median-centered ratio	Prostate gland	Prostate adenocarcinoma	Fold change	P-value	Over-expression Gene Rank
TCGA Prostate	Maximum	0.046	0.407	1.041	8.82E-7	140 (in top 1%)
	90th percentile	0.024	0.13			
	75th percentile	0.017	0.088			
	Median	0.011	0.042			
	25th percentile	0.008	0.025			
	10th percentile	0.005	0.014			
	Minimum	0.001	0.005			
	Number of samples	61	45			

(g) HDAC9

Study	Log2 median-centered ratio	Prostate gland	Prostate carcinoma	Fold change	P-value	Over-expression Gene Rank
Yu Prostate	Maximum	-0.552	1.695	1.278	0.001	832 (in top 10%)
	90th percentile	-0.926	0.669			
	75th percentile	-0.965	-0.684			
	Median	-1.261	-1.137			
	25th percentile	-1.433	-1.321			
	10th percentile	-1.619	-1.591			
	Minimum	-1.732	-2.046			
	Number of samples	23	65			

(h) HDAC10

Study	Log2 median-centered ratio	Prostate gland	Prostate carcinoma	Fold change	P-value	Over-expression Gene Rank
Grasso Prostate	Maximum	0.464	1.152	1.461	2.76E-4	1643 (in top 9%)
	90th percentile	-0.19	0.428			
	75th percentile	-0.411	0.176			
	Median	-0.997	-0.348			
	25th percentile	-1.377	-0.763			
	10th percentile	-1.786	-1.443			
	Minimum	-1.908	-2.543			
	Number of samples	27	59			
